# Investigation of the effect of impacted third molar position and orientation on bad split fractures in sagittal split ramus osteotomy using finite element analysis

**DOI:** 10.1186/s13005-025-00574-x

**Published:** 2025-12-16

**Authors:** İrfan Üstündağ, Erkan Mecu, Yunus Çetiner, Mehmet Sait Şimşek

**Affiliations:** Faculty of Dentistry, Department of Oral and Maxillofacial Surgery, İnönü University, 44280 Campus, Malatya, Turkey

**Keywords:** Bad split, Impacted tooth, Sagittal split ramus osteotomy, Finite element analysis

## Abstract

**Background:**

This study aims to evaluate the impact of different angular orientations and buccolingual positions of impacted third molars on the incidence of bad splits during sagittal split ramus osteotomy using finite element analysis.

**Materials and methods:**

A total of 12 mandibular models were constructed, each representing a unique combination of third molar orientations (vertical, distoangular, horizontal, mesioangular) and positions (buccal, lingual, central). A force of 20 N was applied from the osteotomy line toward the proximal and distal segments to simulate the working principle of the Smith Spreader instrument.

**Results:**

The maximum principal stress followed the order: vertical > distoangular > horizontal > mesioangular. In terms of positional stress distribution, the buccal position generated the highest stress, followed by lingual and central positions. Similar trends were observed across all stress criteria evaluated.

**Conclusion:**

The orientation and position of impacted third molars have a direct effect on the risk of bad splits during SSRO. Mesioangular and centrally positioned teeth were associated with lower stress levels, indicating lower risk, while vertically oriented and buccally or lingually positioned molars showed significantly higher stress concentrations. Thus, extraction of high-risk impacted third molars prior to surgery is recommended to reduce the likelihood of complications.

## Background

Sagittal split ramus osteotomy (SSRO) is considered one of the fundamental procedures in mandibular surgery. It is among the most essential and commonly performed techniques in maxillofacial surgery, offering both aesthetic and functional improvements in the correction of mandibular deformities [1].

SSRO enables repositioning of the mandible by means of strategically placed osteotomies in the mandibular ramus [2]. Initially described by Trauner and Obwegeser, this technique has undergone several modifications aimed at minimizing postoperative complications, notably by Dal Pont [3], Hunsuck [4], and Epker [5]. Despite these improvements, complications such as neurosensory disturbances, bleeding, condylar malposition, and unfavorable fractures—commonly referred to as bad splits—may still occur following SSRO [6, 7].

Although the SSRO technique has evolved with various refinements over time, bad splits continue to pose a significant intraoperative complication [2]. Previous studies have reported the incidence of bad splits to range between 0.21% and 22.72% [8].

Several risk factors have been proposed in relation to the occurrence of bad splits [2, 9]; however, one such factor—the presence of impacted third molars—remains a subject of ongoing debate. The literature presents conflicting findings on whether these teeth contribute to an increased risk of bad splits. For instance, Doucet et al. [10] reported no significant relationship between the presence or removal of impacted third molars and the occurrence of bad splits. Conversely, other studies have suggested that impacted third molars may elevate the risk [11]. Reyneke et al. [12] found that the presence of impacted third molars in patients under the age of 20 was associated with a higher incidence of bad splits. Notably, Balaji et al. [13] were among the first to confirm that the spatial position of impacted third molars could be a contributing factor to the development of bad splits.

While previous studies have examined the influence of sagittal orientations (e.g., mesioangular, distoangular) of third molars on bad split risk, the potential impact of buccal or lingual proximity to the cortical plates has not yet been systematically evaluated.

Surgical procedures performed in the maxillomandibular region have extensively employed three-dimensional finite element analysis (FEA), particularly in applications such as implant placement, zygomatic implants, distraction osteogenesis, and osteotomy procedures [14–19]. Through this method, the biomechanical effects of various surgical approaches have been modeled in detail.

However, to date, no study has comprehensively evaluated the effects of positional variations of impacted third molars on SSRO, taking into account both angular orientation and buccolingual position. Thus, the potential influence of these variations on the occurrence of intraoperative complications during SSRO remains inadequately clarified.

This study aims to fill this critical gap in the literature by analyzing the impact of both the angular orientation and buccolingual position of impacted third molars on the risk of bad splits during SSRO.

Accordingly, we hypothesize that both the angular orientation and the spatial relationship of the third molar to the buccal or lingual cortical plates significantly influence the distribution of stress within the mandible during SSRO, thereby affecting the risk of bad split formation.

In this respect, the study offers a novel contribution by being among the first to comprehensively evaluate this issue using three-dimensional FEA models that integrate both angular and positional parameters.

The findings are expected to provide valuable insights for oral and maxillofacial surgeons in preoperative planning by highlighting the importance of considering third molar presence and position as factors in minimizing surgical complications.

## Methods

This study was conducted through a collaboration between the Faculty of Dentistry at İnönü University and Tinus Technologies. The processes of converting the three-dimensional mesh structure into mathematically suitable solid models, generating and solving FEA models were carried out using HP workstations equipped with an INTEL Xeon E-2286 processor (2.40 GHz) and 64 GB ECC RAM.

### Mandibular bone modeling process

The mandibular bone model was generated using computed tomography data obtained from the Visible Human Project, reconstructed at a slice thickness of 0.33 mm. The DICOM (.dcm) formatted CT data were imported into 3D Slicer software, and segmentation was performed based on appropriate Hounsfield unit thresholds. Following segmentation, three-dimensional models were exported in.stl format.

These models were subsequently imported into Blender software. A 2 mm offset was applied to the outer surface of the mandibular bone to simulate the cortical bone layer, and the trabecular bone was derived from the internal surface of this offset model.

### SSRO modeling and tooth positioning criteria

**Fig. 1:**
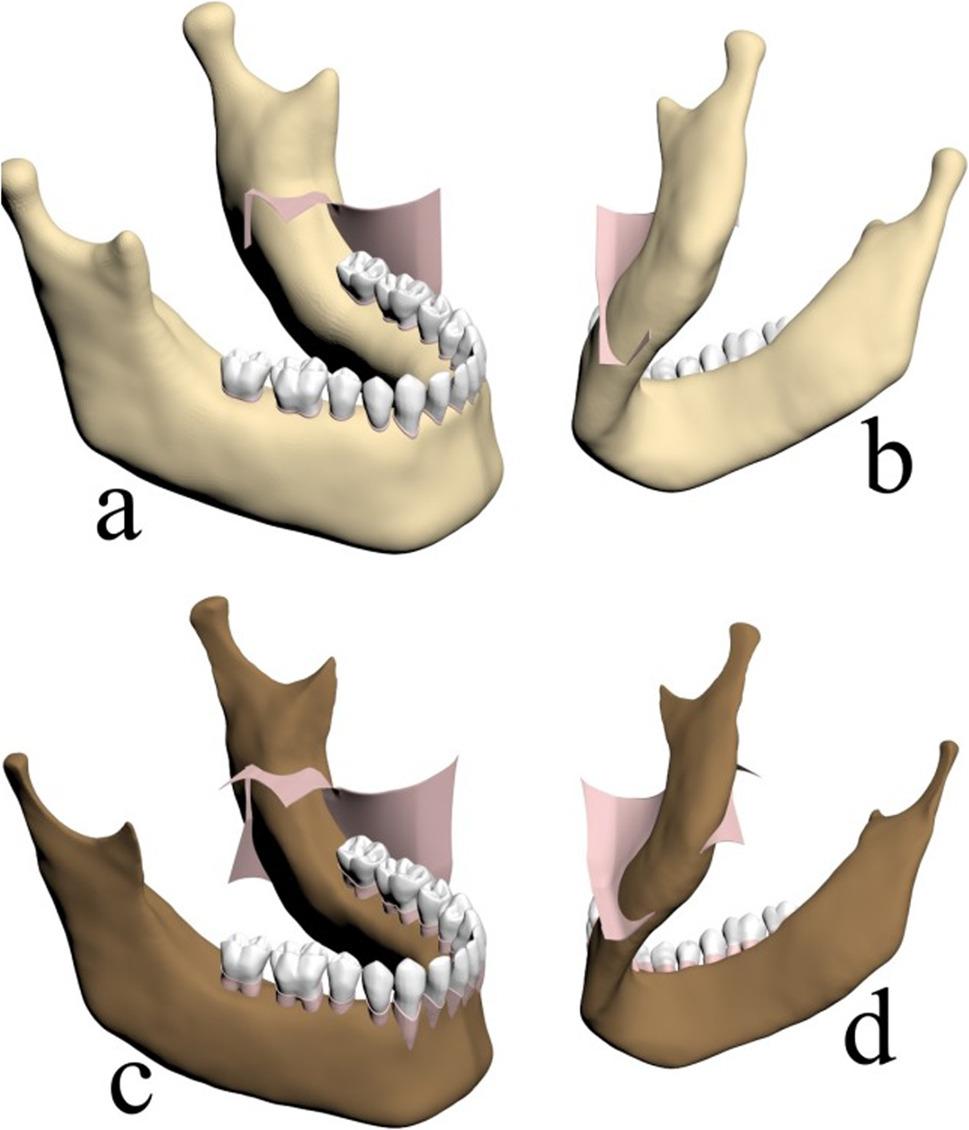
**a** Lingual osteotomy line on the cortical bone, **b** Wolford osteotomy line on the inferior border of the mandibular cortical bone, **c** Osteotomy line created in the trabecular bone, **d** Inferior view of the osteotomy line in the trabecular bone

In this study, a SSRO model was developed. The osteotomy line was designed to extend posteriorly along the lingual surface, starting anterior to the mandibular foramen at the level of the lingula (Figs. 1a and c). Additionally, a 1.5 cm Wolford inferior border osteotomy was created at the midline of the mandibular base (Figs. 1b and d).

The study aimed to investigate the impact of the position of the mandibular third molar (tooth #38) on the occurrence of bad splits during SSRO. The third molars were modeled in four angular orientations based on the classical Winter classification:

- Horizontal
- Mesioangular
- Vertical
- Distoangular [20]

The angular classification was defined according to the angle between the long axis of the third molar and the second molar, as follows:

- Mesioangular: 11–70°
- Horizontal: 71–100°
- Vertical: 0–10°
- Distoangular: >100° [21]

To isolate the effect of positional and angular variations, all models were standardized according to the Pell & Gregory classification as Class 1, Depth C, across the dataset.

The determination of buccal-central-lingual positions was based on the location of the third molar within the coronal plane of the mandibular bone structure:

- Buccal: Positioned closer to the buccal cortical plate
- Central: Located centrally between the buccal and lingual cortices
- Lingual: Positioned closer to the lingual cortical plate

Although the literature lacks a widely accepted classification regarding buccolingual positioning, Qian et al. [22] analyzed differences in alveolar bone thickness according to the buccolingual position of mandibular third molars and provided position-based clinical recommendations. Inspired by this study, the current positional definitions were based on anatomical observation and were consistently applied throughout the modeling process.

Additionally, periodontal ligaments were modeled with a thickness of 0.2 mm, based on the outer surfaces of the teeth.

All models were spatially aligned in three-dimensional space using Blender software, ensuring anatomical accuracy during positioning.

### Generation of mathematical models

**Fig. 2:**
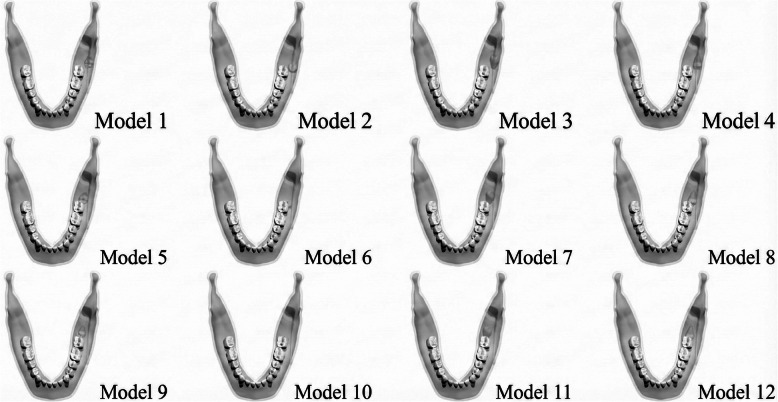
Modeling of the mandibular impacted third molar in different positions

Based on predetermined osteotomy lines and measurements applied to the mandibular cortical and trabecular bone, 12 primary models were generated in this study (Table 1; Fig. 2).

**Table 1:** Modeling of the mandibular impacted third molar in different positions

Models	Model Characteristics
Model 1	Impacted tooth in buccal position - mesioangular orientation
Model 2	Impacted tooth in buccal position - vertical orientation
Model 3	Impacted tooth in buccal position - distoangular orientation
Model 4	Impacted tooth in buccal position - horizontal orientation
Model 5	Impacted tooth in central position - mesioangular orientation
Model 6	Impacted tooth in central position - vertical orientation
Model 7	Impacted tooth in central position - distoangular orientation
Model 8	Impacted tooth in central position - horizontal orientation
Model 9	Impacted tooth in lingual position - mesioangular orientation
Model 10	Impacted tooth in lingual position - vertical orientation
Model 11	Impacted tooth in lingual position - distoangular orientation
Model 12	Impacted tooth in lingual position - horizontal orientation

**Fig. 3:**
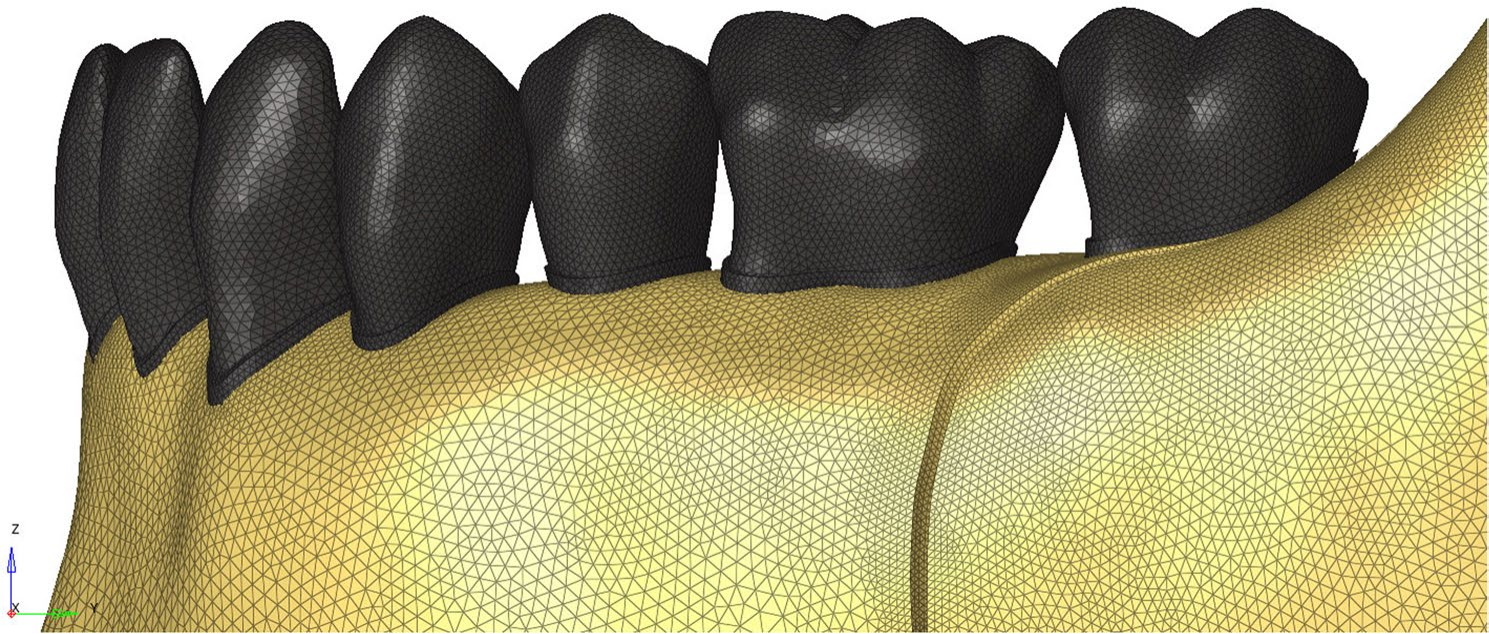
Subdivision of geometric models into meshes

The geometric models were converted into mathematical models by subdividing them into small and simple mesh elements (Fig. 3). After completing the modeling in Blender, ALTAIR HyperMesh software was used to generate the mathematical mesh structures. In this study, high-precision triangular (tria) mesh elements with sizes ranging between 0.1 and 0.25 mm were used.

After covering the surface of each model with triangular mesh elements, the volumetric structures were meshed using tetrahedral (regular four-faced) elements. The resulting mathematical models were then imported into the ALTAIR OptiStruct solver for FEA.

### Material definitions

The anatomical structures used in the models were defined with linear elastic material properties. Mechanical properties such as elastic modulus and Poisson’s ratio were numerically assigned and used in the analysis (Table 2).

**Table 2:** Linear material properties of materials with given elastic modulus and poisson’s ratio

Material	Elastic Modulus [MPa]	Poisson’s Ratio
Cortical Bone	13,700	0.30
Trabecular Bone	1,370	0.30
Tooth	18,600	0.31
Periodontal Ligament	50	0.45

### Loading scenarios and boundary conditions

**Fig. 4:**
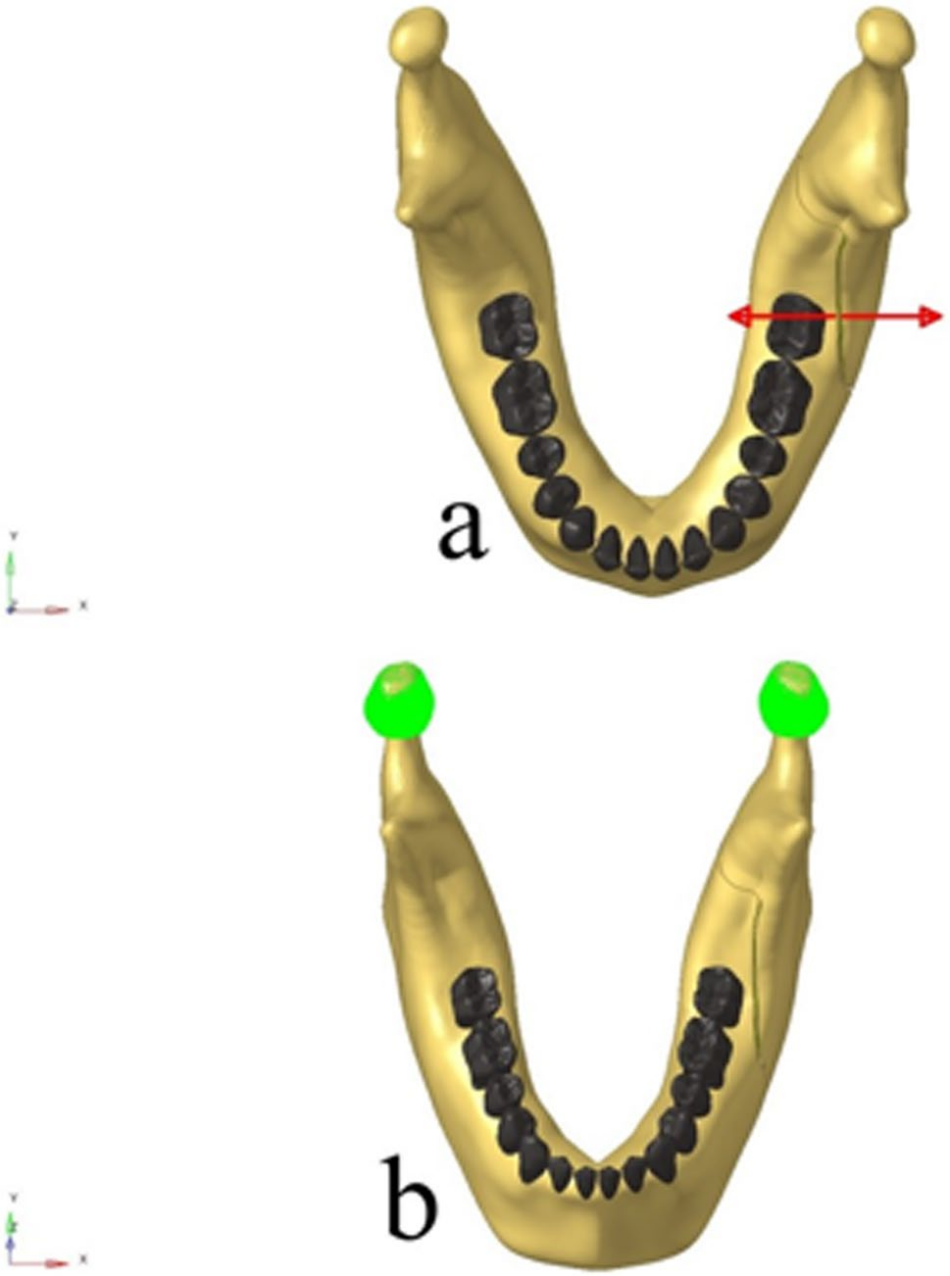
**a** Schematic illustration of the simulation of the Smith Spreader hand instrument applying a 20 N force, **b** Schematic illustration of boundary conditions showing fixation by constraining all degrees of freedom at the nodal points in the condylar regions, preventing movement along all three axes

A 20 N force was applied to the trabecular bone, directed between the proximal and distal segments across the osteotomy site in all models. This magnitude was selected based on literature as a representative value simulating the average surgical force exerted by the Smith Spreader hand instrument [23, 24]. The force direction and point of application were designed to replicate the instrument’s operational principle (Fig. 4a).

To simulate surgical stabilization, all degrees of freedom (6 DoF) at the nodal points of the condylar regions of the mandible were constrained, restricting motion in all three spatial axes (Fig. 4b). This boundary condition reflects the passive stabilization of the mandible during surgery through muscle relaxation and intraoperative manipulation.

Under these loading and boundary conditions, a total of 12 linear static analyses were performed—one for each primary model (Fig. 2).

The FEA models used in this study were designed in accordance with previously published parameters in similar maxillofacial finite element studies, ensuring compatibility in terms of anatomical fidelity, osteotomy design, loading conditions, and boundary constraints [23, 25].

### Quantitative model information

The numerical data for all 12 generated models are presented in Table 3.

**Table 3:** Constructed models

Model	Total Number of Nodes	Total Number of Elements
Model 1	913,338	3,586,093
Model 2	898,279	3,516,411
Model 3	901,808	3,535,288
Model 4	906,428	3,554,841
Model 5	867,116	3,398,968
Model 6	874,360	3,425,490
Model 7	874,803	3,432,127
Model 8	885,205	3,479,595
Model 9	956,107	3,758,267
Model 10	894,189	3,499,505
Model 11	903,978	3,544,138
Model 12	906,951	3,556,827

#### Assembly of components and surface interactions

To ensure accurate results during analysis, surface interactions between all model components were explicitly defined within the simulation software. For this purpose, BONDED-type contact definitions were applied to all interaction surfaces. This type of contact assumes that the connected components will behave as a fully integrated unit, without any relative motion occurring between them during the simulation.

## Results

FEA revealed that both the positional placement and angular orientation of impacted third molars caused significant variations in stress distribution within the mandibular region. The highest stress values were observed in the buccal position, while the lowest stress levels were detected in the central position.

### Evaluation based on angular orientation

**Fig. 5:**
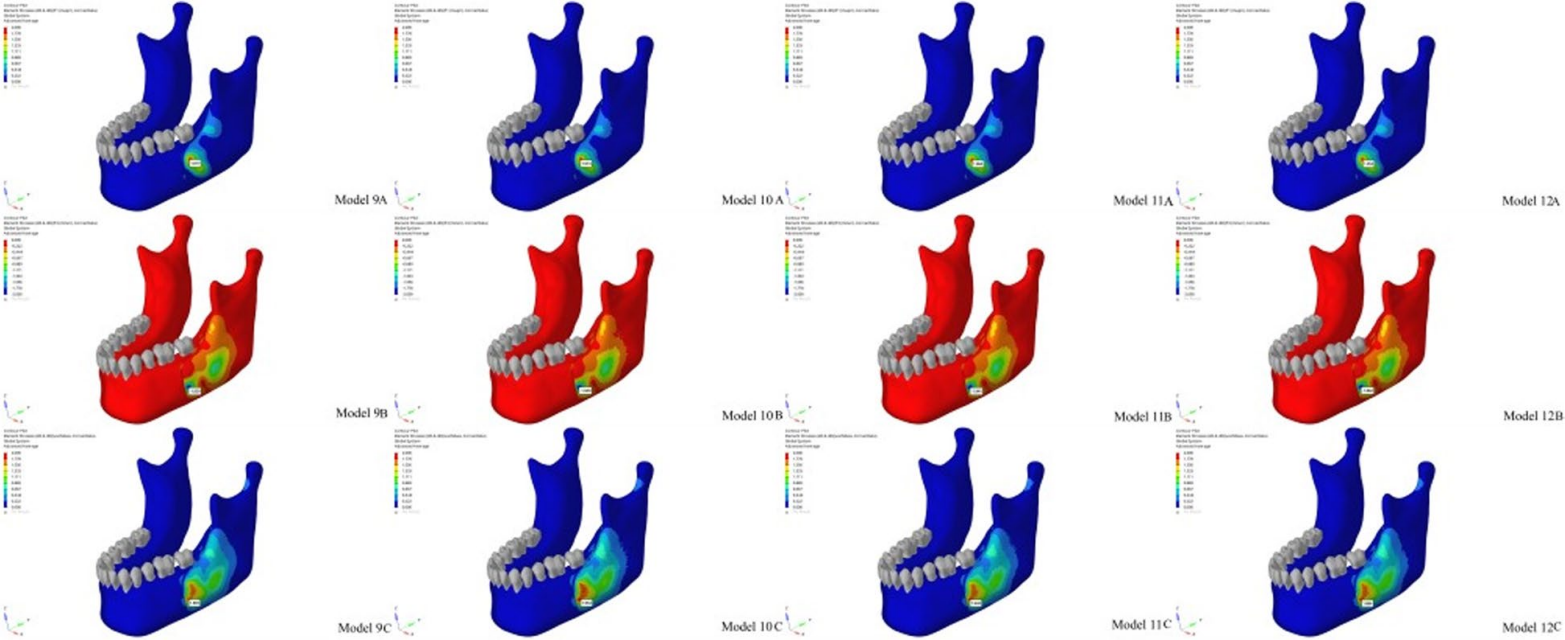
9A: Minimum Principal stress value of impacted tooth in lingual position with mesioangular orientation, 9B: Maximum Principal stress value of impacted tooth in lingual position with mesioangular orientation, 9C: Von Mises stress value of impacted tooth in lingual position with mesioangular orientation, 10A: Minimum Principal stress value of impacted tooth in lingual position with vertical orientation, 10B: Maximum Principal stress value of impacted tooth in lingual position with vertical orientation, 10C: Von Mises stress value of impacted tooth in lingual position with vertical orientation, 11A: Minimum Principal stress value of impacted tooth in lingual position with distoangular orientation, 11B: Maximum Principal stress value of impacted tooth in lingual position with distoangular orientation, 11C: Von Mises stress value of impacted tooth in lingual position with distoangular orientation, 12A: Minimum Principal stress value of impacted tooth in lingual position with horizontal orientation, 12B: Maximum Principal stress value of impacted tooth in lingual position with horizontal orientation, 12C: Von Mises stress value of impacted tooth in lingual position with horizontal orientation

Considering all stress criteria (Maximum Principal, Minimum Principal, and von Mises), the average stress rankings were as follows (Fig. 5):

- Vertical > Distoangular > Horizontal > Mesioangular

The vertical orientation generated approximately 30% higher stress compared to the mesioangular position.

The distoangular orientation produced roughly 25% more stress than mesioangular.

The horizontal orientation resulted in about 20% higher stress than mesioangular.

Among all angular positions and buccolingual locations, the mesioangular orientation consistently exhibited the lowest stress levels.

This trend was observed consistently across all evaluated stress parameters (Table 4).

**Table 4:** Values of all analyzed stress criteria

Impacted Tooth Orientation	Impacted Tooth Position	Stress Region	Maximum Principal [MPa]	Minimum Principal [MPa]	Von Mises [MPa]
Mesioangular	Buccal	Buccal	1.784	−1.705	1.845
		Lingual	0.184	−0.342	0.307
Vertical	Buccal	Buccal	1.863	−1.888	1.940
		Lingual	0.224	−0.410	0.358
Distoangular	Buccal	Buccal	1.851	−1.857	1.930
		Lingual	0.206	−0.377	0.338
Horizontal	Buccal	Buccal	1.819	−1.728	1.860
		Lingual	0.197	−0.361	0.334
Mesioangular	Middle	Buccal	1.446	−1.681	1.717
		Lingual	0.165	−0.335	0.283
Vertical	Middle	Buccal	1.581	−1.770	1.874
		Lingual	0.189	−0.371	0.328
Distoangular	Middle	Buccal	1.566	−1.742	1.834
		Lingual	0.181	−0.362	0.315
Horizontal	Middle	Buccal	1.482	−1.721	1.830
		Lingual	0.171	−0.345	0.302
Mesioangular	Lingual	Buccal	1.817	−1.839	1.865
		Lingual	0.225	−0.388	0.326
Vertical	Lingual	Buccal	1.913	−1.948	1.964
		Lingual	0.253	−0.445	0.360
Distoangular	Lingual	Buccal	1.864	−1.940	1.940
		Lingual	0.248	−0.399	0.353
Horizontal	Lingual	Buccal	1.853	−1.884	1.884
		Lingual	0.233	−0.391	0.343

### Positional differences

Stress distribution based on the buccolingual position of the third molar followed the order:

- Buccal > Lingual > Central

The buccal position produced the highest stress values across all angular orientations.

Although anatomically located further from the buccal plate, lingually positioned molars still induced 5–10% higher stress on the buccal cortical plate.

The central position demonstrated 20–30% lower stress levels across all positional and angular variations, highlighting it as the most favorable location in terms of stress reduction.

### Comparative findings

A vertically oriented third molar in the buccal position generated approximately 35% more stress compared to a mesioangular tooth in the buccal position.

A distoangular third molar in the buccal position produced 30% higher stress than the mesioangular configuration.

A vertically oriented tooth in the lingual position induced approximately 20% more stress than a distoangular tooth.

The mesioangular tooth in the central position consistently demonstrated the lowest stress levels, with 30–35% less stress than all other positional and angular combinations, thus representing the lowest-risk model.

### Fracture direction prediction

The vertical concentration of stress observed in the buccal cortical plate suggests that potential fractures in this region are likely to propagate in a vertical direction.

In contrast, the horizontal distribution of stress observed in the lingual cortical plate indicates a tendency for potential fractures to occur along the horizontal axis in this area.

## Discussion

In this study, the influence of the position and angular orientation of impacted mandibular third molars on stress distribution during SSRO was evaluated using the FEA method. The findings indicate that both positional placement (buccal, central, lingual) and angular orientation (mesioangular, distoangular, vertical, horizontal) of the tooth are significant determinants in the likelihood of bad splits.

The mesioangular orientation, where the crown of the third molar tilts toward the second molar, is the most commonly encountered impaction type among all third molar orientations [26]. In this configuration, the roots are relatively distant from the osteotomy line, which reduces stress concentration within the surrounding bone. According to FEA analyses, the mesioangular orientation consistently generated the lowest stress values across all positions (buccal, lingual, and central). Specifically, mesioangular teeth located in the central position produced approximately 35% lower maximum principal stress compared to vertically impacted teeth in the same location. This reduced stress distribution contributes to a smoother osteotomy line and a lower risk of bad split occurrence. Similarly, previous studies have reported that mesioangular third molars present fewer surgical complications compared to other impaction types [23, 25].

In the distoangular orientation, the crown of the third molar is tilted distally, while the roots are directed anteriorly. This oblique configuration causes asymmetric stress distribution along the osteotomy line. FEA analyses showed that the distoangular model generated approximately 30% higher von Mises stress compared to the mesioangular model in the same position. This increase in stress compromises the formation of a smooth fracture line and leads to localized stress accumulation. These findings are consistent with the observations by Kim et al. [27], who reported longer surgical durations and a higher incidence of cortical fractures in SSRO procedures involving distoangular third molars.

Third molars in the vertical orientation are often deeply embedded within the bone and tend to have multiple roots, contributing to significant morphological and volumetric resistance along the osteotomy pathway. Among all angular positions evaluated, the vertical position exhibited the highest stress values in the FEA analyses. In particular, vertically positioned third molars located buccally produced approximately 35% higher maximum principal stress than their mesioangular counterparts in the same location. This elevated stress is attributed to reduced bone elasticity and the concentration of force in a narrow region. Supporting this, Bingül et al. [28] reported that vertical impactions are both the most commonly observed and the most complication-prone form of third molar impaction, in line with the results of the present study.

The horizontal orientation, in which the crown of the third molar is positioned anteriorly in the horizontal plane, is considered one of the most surgically challenging configurations. In this alignment, the tooth extends transversely along the mandible, forming the largest volumetric structure intersecting the osteotomy line. FEA analyses revealed that horizontally impacted third molars generated approximately 25% higher von Mises stress than mesioangular molars in the same position. This elevated stress contributes to deviations in the osteotomy pathway, making it more difficult to establish a controlled fracture line. These findings support the importance of precise osteotomy planning, as emphasized by Epker [5]. Furthermore, the horizontal orientation was found to concentrate stress in the lingual cortical plate, particularly in the horizontal axis, suggesting a potential association with lingual segment fractures [29].

Not only the angular orientation of the third molar, but also its positional placement (buccal, central, or lingual) has a significant effect on stress distribution. According to our findings, the buccal position consistently produced the highest stress values across all angular orientations. The primary reason for this appears to be the thinner structure of the buccal cortical plate and its direct exposure to external surgical forces. This observation is consistent with the findings of Gumber et al. [30], who reported that cortical bone thickness varies according to third molar position, influencing both surgical difficulty and stress concentration patterns.

During spreader maneuvers, the presence of vertically concentrated stress in the buccal cortical plate was found to significantly increase the risk of fracture. Similarly, Derin et al. [23] identified vertical stress accumulation in the buccal cortex as a primary biomechanical mechanism contributing to the formation of bad splits. Although total stress values were lower in the lingual position, the reduced thickness of the lingual cortical bone was found to facilitate the transformation of horizontal stress into fracture. This observation is consistent with the findings of Wolford et al. [31], who reported a higher incidence of lingual segment fractures under similar conditions.

In centrally positioned third molars, the equidistant placement from both buccal and lingual plates allows for a more balanced internal stress distribution, thereby minimizing the risk of fracture. The results of this study align well with the existing literature. Specifically, the finite element modeling parameters we adopted are methodologically consistent with the criteria outlined by Shyam Sundar et al. [25] and Al-Rafee et al. [32].

Nevertheless, our findings diverge from those reported by Jiang et al. [33], who claimed that the presence of impacted third molars does not significantly increase the risk of bad splits. In contrast, our analysis suggests that the position and orientation of the third molar play a critical role in determining the fracture risk during SSRO procedures.

Fixation along the Champy line is essential for maintaining postoperative stability following SSRO. However, when impacted third molars are located beneath the external oblique ridge (EOR), it may impede screw placement and compromise fixation stability [13, 34]. For this reason, in our study, the third molars were modeled below the EOR to simulate a clinically high-risk fixation scenario.

Moreover, applying the spreader before completing the osteotomy line may result in uncontrolled fracture patterns. Previous studies have reported that directing the spreader force closer to the inferior border of the mandible, away from the third molar, and in a more anterior or inferior direction can significantly reduce the risk of bad splits [35]. Our findings also demonstrated that in vertically and distoangularly impacted molars located buccally, inappropriate loading directions significantly increased stress concentration, thereby escalating the risk of fracture.

From a clinical perspective, the preoperative extraction of impacted third molars that are located in high-stress orientations is strongly recommended. This approach not only enhances segmental stability but also reduces operative time and lowers the risk of postoperative complications [36, 37]. Furthermore, during osteotomy planning, multiple parameters—including the relationship of the tooth to the external oblique ridge (EOR), cortical thickness, and the direction of spreader force application—should be carefully evaluated in combination.

FEA method provides a powerful computational framework for assessing stress distributions in the mandibular bone. In the present study, the 20 N force applied was based on the average clinical force exerted by the Smith Spreader device during SSRO procedures [23, 24]. This magnitude is consistent with the typical spring forces used during mandibular osteotomies, as reported in the literature.

However, it should be acknowledged that FEA models are based on idealized geometries and assume homogeneous material properties, and in this study, the modeling data were derived from cone-beam computed tomography (CBCT) scans [38]. As such, biological variables such as individual bone density, age, sex, and viscoelastic properties of soft tissues cannot be fully incorporated into the analysis [32]. Nevertheless, despite these limitations, FEA remains a clinically valuable tool for comparing stress patterns and predicting high-risk fracture regions in a systematic and reproducible manner [25].

Based on the findings of this study, the following surgical considerations are recommended for optimal SSRO planning:

Mesioangular and centrally positioned third molars are biomechanically safer and do not significantly complicate the osteotomy procedure.

In contrast, vertically or distoangularly oriented impacted third molars, particularly in the buccal or lingual positions, should be carefully evaluated and considered for extraction prior to SSRO.

During spreader application, forces should be applied closer to the mandibular inferior border, away from the tooth, and distributed symmetrically to avoid stress imbalance. Sudden or asymmetrical forces should be strictly avoided.

## Conclusion

This study demonstrated that the angular orientation and buccolingual position of impacted third molars significantly influence the stress distribution in the mandible during SSRO.

According to the FEA results, mesioangular and centrally positioned molars produced the lowest stress values, averaging 8.5–9.2 MPa, whereas vertical and distoangular orientations, particularly in the buccal position, generated approximately 30–35% higher stress levels (11.8–12.4 MPa).

These findings indicate that the risk of bad split fractures during SSRO is directly associated with the position and orientation of the third molar.

From a clinical perspective, the preoperative extraction of impacted third molars—other than those in mesioangular and central positions—may reduce stress accumulation along the osteotomy line and thereby minimize fracture risk.

Furthermore, the application of spreader forces symmetrically and close to the mandibular inferior border should be considered a critical factor in preventing uncontrolled fracture lines.

This study emphasizes the importance of third molar evaluation during SSRO planning to enhance surgical safety and predictability.

However, to strengthen the clinical relevance of these biomechanical findings, future in vivo and prospective clinical studies are warranted.

## Data Availability

The datasets generated and/or analyzed during the current study are available from the corresponding author on reasonable request.
